# Buddy taping after reduction of displaced extra-articular phalangeal finger fractures in children: a randomized controlled trial

**DOI:** 10.1177/17531934241293338

**Published:** 2024-11-02

**Authors:** Daniel M. Weber, Christian Luckert, Markus Kalisch, Ulrike Subotic, Robert Weil, Michelle Seiler

**Affiliations:** 1 Division of Hand Surgery, University Children’s Hospital Zurich, Switzerland; 2 Children’s Research Centre, University Children’s Hospital Zurich, University of Zurich, Switzerland; 3 Seminar for Statistics, ETH Zurich, Switzerland; 4 Department of Paediatric Surgery, University Children’s Hospital Basel, Switzerland; 5 Department of Paediatric Surgery, Hospital Baden, Switzerland; 6 Paediatric Emergency Department, University Children’s Hospital Zurich, Switzerland

**Keywords:** Children, finger fracture, phalangeal fractures, treatment

## Abstract

In this randomized controlled trial, we assessed the non-inferiority of buddy taping to splinting after reduction of displaced extra-articular proximal and middle phalangeal finger fractures in children. The primary outcome was the rate of secondary fracture displacements; the secondary outcomes were patient comfort, analgesic efficacy and total range of active motion 6 months after injury. Eighty-one patients participated: 43 with taping and 38 with splinting. Secondary displacement occurred in eight patients: five in the splinting group and three in the taping group. Risk difference was below the predefined non-inferiority of 10%. Patient comfort was significantly higher in the taping group, with no group differences for other parameters. Our previous study recommended taping for undisplaced finger fractures in children. With the current data, we recommend taping these finger fractures irrespective of displacement or need for reduction. We are encouraged to propose taping as an alternative to splinting for increased patient comfort, lower cost, and shorter application time.

**Level of evidence:** I

## Introduction

The treatment of finger fractures in children has become more conservative with less rigid immobilization over recent years. Buddy taping is an efficient treatment for undisplaced, stable finger fractures in children (Nellans and Chung, 2013; Weber et al., 2019). Patient satisfaction is high, and the risk of secondary displacement is minimal, similar to forearm-based splint immobilization (Weber et al., 2019).

Treatment strategies for extra-articular displaced finger fractures in children have changed in recent decades from a predominantly surgical approach (Abzug and Zlotolow, 2012; Belsky et al., 1984) to a more conservative treatment with closed reduction and cast immobilization (Bergeron, 2005; Boyer et al., 2015; Cornwall and Ricchetti, 2006; Franz et al., 2012; Kozin et al., 2000; Verver et al., 2017).

Fracture immobilization with buddy taping offers several advantages over splinting (Weber et al., 2019; Won et al., 2014). A child is less restricted in daily activities, treatment costs are lower and application time is significantly shorter (Weber et al., 2019). Until recently, buddy taping has only been recommended for undisplaced, length-stable fractures in children (Nellans, 2013). In a previous study, we provided evidence to support this recommendation by demonstrating that buddy taping is not inferior to cast immobilization for treating undisplaced phalangeal fractures (Weber et al., 2019). Although that study included patients with displaced phalangeal fractures, the sample size remained insufficient to conclusively determine outcomes for this specific subgroup. Hence, we conducted the present study to assess whether buddy taping is not inferior to cast immobilization for treating displaced extra-articular phalangeal fractures following closed reduction.

## Methods

This prospective, randomized, single-centre study was conducted at the University Children’s Hospital, Zurich, between August 2019 and January 2023. Patients were eligible for study inclusion if aged 4–16 years and presenting at the emergency department with displaced phalangeal fractures requiring closed reduction. Exclusion criteria comprised fractures of the distal phalanges, multiple fractures in the same hand, open fractures, intra-articular fractures, phalangeal neck fractures, fractures with multiple fragments and presentations delayed more than 5 days after the accident. Long oblique fractures were included and regarded as unstable fractures.

Ethical approval for this study was provided by the local ethics committee. Written informed consent was obtained from all parents and patients aged 14 years and older, and verbal informed consent was obtained from younger patients.

The study design was identical to our previous study, in which the same fracture types were included irrespective of the degree of displacement (Weber et al., 2019). Patient numbers were too small for a subgroup analysis of displaced fractures that were reduced before immobilization in the previous series, which was performed between October 2011 and March 2016. We therefore decided to add the data of patients from the subgroup of displaced fractures from the previous study to the data of the present study to include a total of 81 patients, in order to provide sufficient numbers according to the *a priori* power calculations (see Supplemental Figure S1). We therefore followed a pre-defined strict fixed-sequence procedure for hierarchically ordered correlated multiple endpoints in clinical trials (Huque and Alosh, 2008).

### Interventions

Patient assessment included both clinical examination and radiographic evaluation. Criteria for closed fracture reduction included rotational deformity or an angulation greater than 10° in the frontal plane or greater than 25° in the sagittal plane. Closed reductions were performed by paediatric emergency physicians while patients inhaled nitrous oxide. The method of fracture stabilization was randomized prior to closed fracture reduction. A list assigning patients randomly to either buddy taping or splinting in a 1:1 ratio was generated by computer and the allocations were sealed in envelopes. Each patient selected an envelope specifying the treatment to which they had been allocated. Fractures were immobilized for 3 weeks.

**Figure 1.**
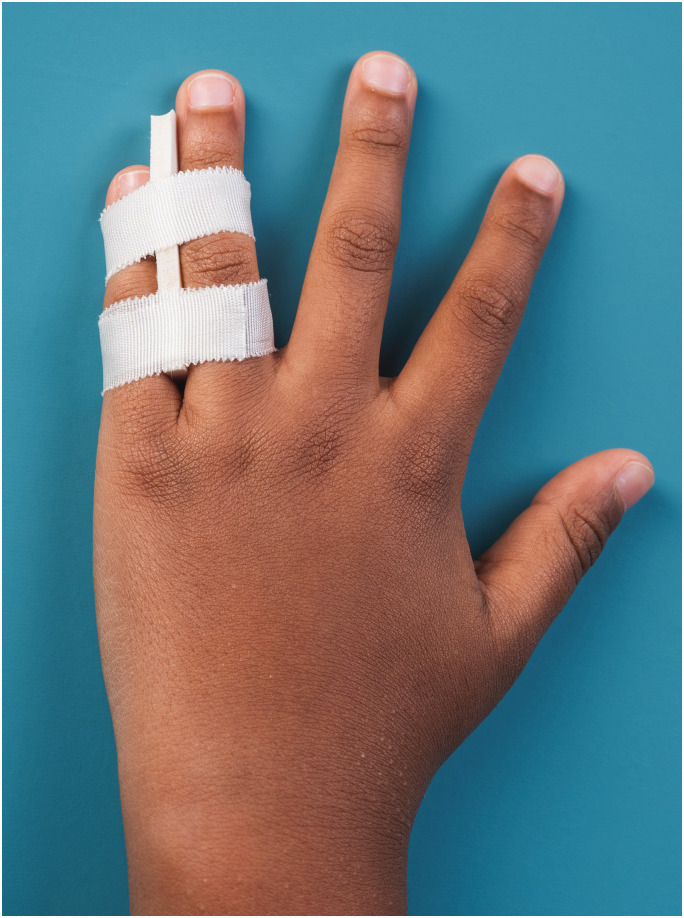
Finger taping with interdigital padding.

*Taping* – The fractured finger was taped to its neighbouring finger using a standard tape (Strappal; BSN Medical Ltd, Willerby, UK) with interdigital padding (Leukotape foam; BSN Medical Ltd) (Figure 1). Families received detailed instructions on how to change the taping if desired due to loosening or hygiene.

**Figure 2.**
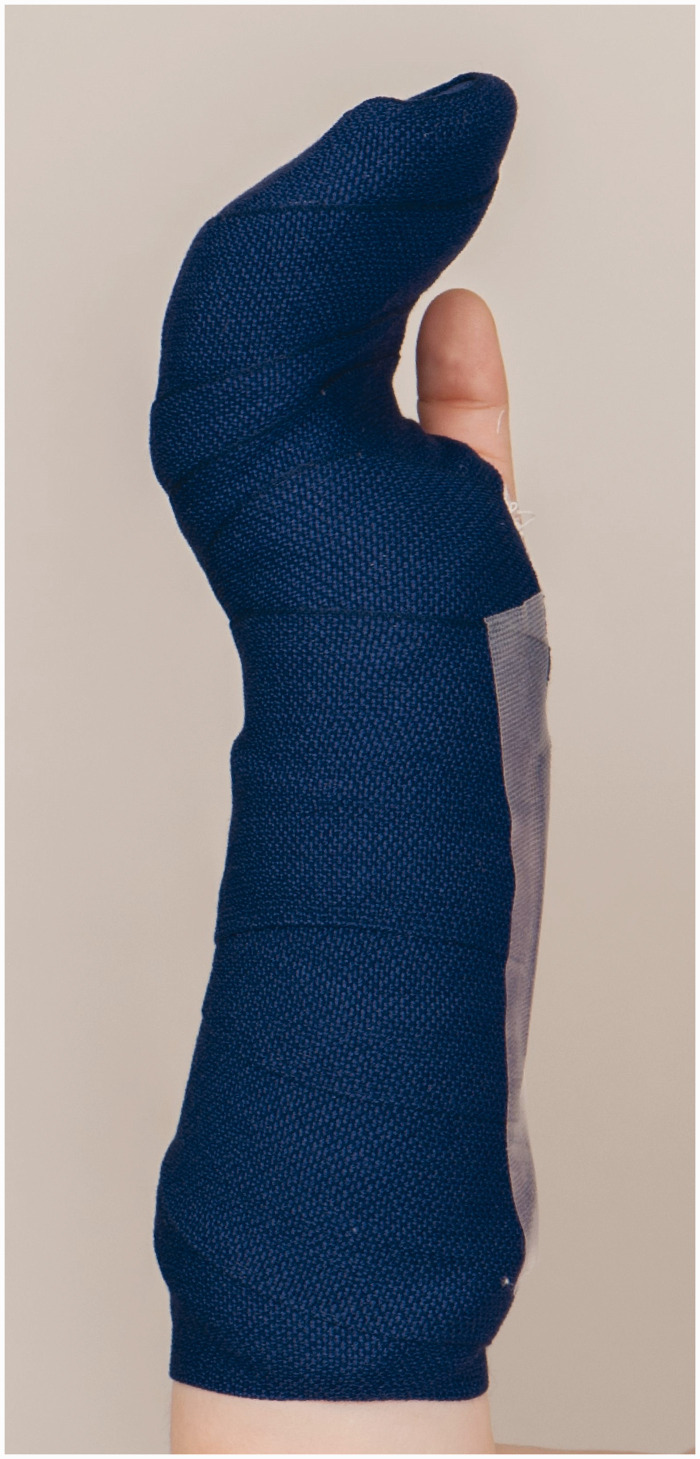
Forearm-based palmar hand splint.

*Splinting* – A forearm-based palmar hand splint was used to include all fingers in an intrinsic plus position (Figure 2). A knitted fibreglass substrate impregnated with a polyurethane resin was used (Scotchcast; 3M, Ruschlikon, Switzerland).

Physicians, parents and patients were not blinded, but the data analyst remained blinded to the treatment groups while reviewing radiographs for the degree of displacement and doing the analyses.

### Outcome measures

**Figure 3.**
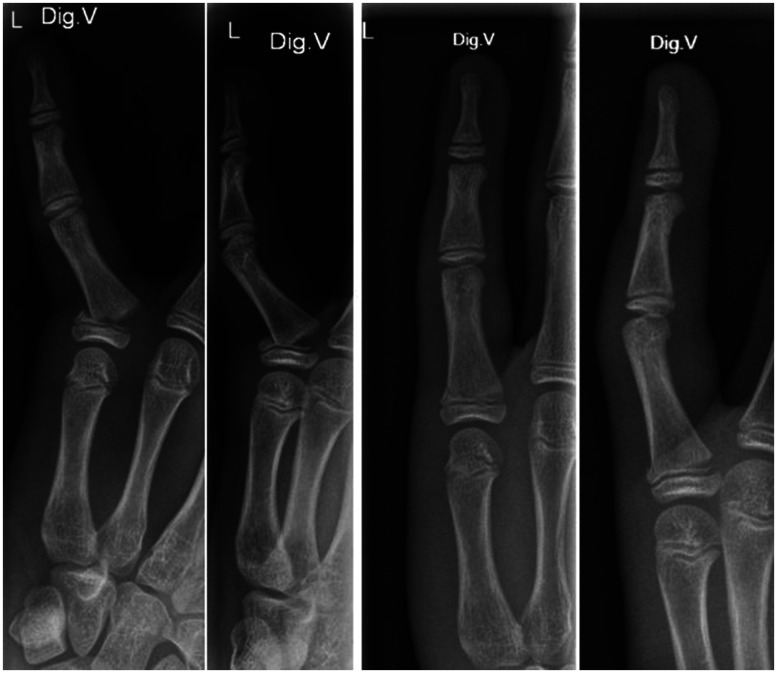
Example of a juxta-epiphyseal fracture of the proximal phalanx of the little finger treated with buddy taping. Posteroanterior and lateral radiographs at initial presentation and after 21 days.

The primary outcome measure was the rate of secondary fracture displacement, which was assessed clinically by hand surgeons and measured from posteroanterior and lateral radiographs on days 5 and 21 (Figure 3; see also Supplemental Figures S2 and S3). Redisplacement was defined as angulation greater than 10° in the radioulnar plane or angulation greater than 25° in the dorsopalmar plane or a rotational deformity of the finger.

The secondary outcome measures included patient comfort and total range of active motion (TRAM) 6 months post-injury. Patient comfort was assessed with a visual analogue scale from 0 (not disturbed at all) to 10 (very disturbing) on days 5 and 21. Additionally, the duration of pain medication required in days was assessed by asking parents during the follow-up visits at days 5 and 21.

### Sample size

In our *a priori* sample size calculation, we assumed that a risk difference of up to 10% with respect to secondary displacement was clinically insignificant. With this criterion and the assumption of a –10% risk difference with taping, enrolling 40 patients in each group allows us to establish non-inferiority with a power of 96% (Monte-Carlo simulation using 10,000 simulations with displacement probabilities of 1% and 11%). Considering the 31 patients with displaced fractures included in our previous study, we decided to include additional patients with a randomized assignment to the groups until a total of 81 patients was reached.

### Statistical analysis

Continuous data are displayed as means and 95% CIs. Categorical data are shown in tables. The primary outcome (i.e. the number of secondary displacements within 5 days of treatment) was analysed with Newcombe’s method 10 (Newcombe, 1998). For the secondary outcomes, differences between groups were analysed with the two-sample *t*-test for continuous data and Fisher’s exact test for categorical data (based on simulation with 100,000 runs for the variable ‘Digit’ because the related table has more than two rows). To deal with values missing from the secondary outcomes, we used multiple imputation (Rubin, 2004) to create and analyse 50 multiply imputed datasets. Incomplete variables were imputed under fully conditional specification (van Buuren et al., 2006). Model parameters were estimated with two-sided, two-sample *t*-tests applied to each imputed dataset separately. These estimates and their standard errors were combined using Rubin’s rules.

## Results

Eightly-one patients were included: 43 randomized to the taping group and 38 to the splinting group (see Supplemental Figure S1, study flowchart and supplementary CONSORT (Consolidated Standards of Reporting Trails) checklist). Patient characteristics, including age, sex, fracture localization and type, were similar between the groups (Table 1).

**Table 1.** Patient characteristics in the two groups.

Baseline data	Taping	Splinting	*p*-value (statistical test)
Patients (*n*)	43	38	
Boys/girls	24/19	25/13	0.374 (Fisher)
Age (median (range)) (years)	11 (3.5-15)	11 (4.5-14)	0.769 (Wilcox)
Fracture side: left/right (*n*)	21/22	25/13	0.177 (Fisher)
Digit: II/III/IV/V (*n*)	0/3/4/36	1/3/5/29	0.763 (Fisher)
Phalanx: proximal/middle (*n*)	40/3	36/2	1.000 (Fisher)
Base/shaft (*n*)	39/4	38/0	0.119 (Fisher)

II = index finger; III: middle finger; IV: ring finger; V: little finger.

Two patients underwent premature study termination: one 7-year-old girl whose parents declined the final visit for TRAM documentation, and one 11-year-old boy who insisted on changing from splinting to taping at the first follow-up visit. The primary outcome could be measured in both cases, and missing values were introduced in the secondary outcomes. In both cases, the injuries healed without any complications. While there were no missing values in the primary outcome, 15 patients had at least one missing value among the four variables of the secondary outcomes (number of missing observations per variable: ‘Comfort after 5 days (VAS)’ had four missing observations, ‘Comfort after 21 days (VAS)’ had nine missing observations, ‘Analgesia intake (days)’ had five missing observations, ‘TRAM (degrees)’ had 12 missing observations).

### Primary outcome

Five of the 38 patients (13%) in the splinting group and three of the 43 patients (7%) in the taping group had secondary displacements of the reduced fractures. The risk difference *p* between the taping and the splinting groups (*p_taping – p_splinting*) for all fractures was –6% with a 95% CI (–21.1% to 7.6%). Therefore, we can conclude that taping is not inferior to splinting in our patients when we assume that an increase of up to 10% in the probability of secondary displacement is clinically irrelevant.

Secondary fracture displacements occurred only in fractures of the base of the proximal phalanges of the little finger, and affected five boys and three girls.

The management of the secondary displaced fractures required intervention in four out of eight cases, with two patients in each group needing surgery or another closed reduction. Closed reduction and K-wire immobilization was performed in two patients, 6 and 8 years old, with secondary displacement of juxta-epiphyseal fractures of the proximal phalanges of the little fingers: radial and dorsal in the taping group and ulnar and dorsal in the splinting group. In the remaining four cases, minimal displacements were considered clinically irrelevant, and new splinting was administered or an additional follow-up with repeat radiographs was conducted. The outcomes were excellent in all cases, which we attributed to the high potential for correction in base fractures in children.

The most common fracture types were base fractures: 59 juxta-epiphyseal fractures (Figure 3) and 12 Salter–Harris II fractures (see Supplemental Figure S2). The latter were treated with taping and splinting in six cases each, and no secondary fracture displacements were observed. By contrast, juxta-epiphyseal fractures accounted for the eight secondary fracture displacements, of which three had been treated with taping and five with splinting.

### Secondary outcomes

Self-reported patient comfort after 5 and 21 days was significantly higher in the taping group than in the splinting group (Table 2). Duration of analgesic intake did not differ significantly between the groups. Furthermore, the assessment of TRAM displayed normal values in all patients with no significant group differences.

**Table 2.** Secondary outcomes.

Measurements	Taping	Splinting	*p*-value	95% CI
Comfort after 5 days (VAS)	1.4	3	0.002	−2.7 to −0.6
Comfort after 21 days (VAS)	0.9	2.2	0.025	−2.1 to −0.1
Analgesia intake (days)	1.9	2.2	0.529	−1.4 to 0.7
TRAM (degrees)	287	292	0.387	−15.7 to 6.2

Data are presented as means.

TRAM: total range of active motion; VAS: visual analogue scale from 0 (not disturbed at all) to 10 (very disturbing).

## Discussion

Fracture immobilization with buddy taping is a valid treatment option for extra-articular displaced proximal and middle phalangeal finger fractures in children following closed reduction. Taping is non-inferior to splinting in preventing secondary fracture displacement and notably enhances comfort levels.

A risk factor for secondary fracture displacement was the location of the fracture within the phalanx with a higher risk of juxta-epiphyseal fractures than Salter–Harris II fractures. Both splinting and taping immobilized these fractures sufficiently in most cases, but a secondary fracture displacement was observed in eight cases (14%). Some studies assume that juxta-epiphyseal fractures are prone to severe displacement and instability after closed reduction and therefore recommend primary surgery with K-wire immobilization (Stash et al., 2022). However, our findings indicate that only eight out of 59 of these fractures redisplaced, whether they were taped or splinted. Therefore, non-surgical treatment with splinting or taping seems to be a justified treatment approach, considering the relatively low secondary displacement rate at follow-up radiographs.

Most finger fractures affect the little finger, particularly the proximal phalanges (Al-Qattan et al., 2008; Hastings and Simmons, 1984; Liu et al., 2014; Vonlanthen et al., 2019). In our cohort, 65/81 (80%) of the fractures involved the little finger and, in 76/81 (94%), the proximal phalanx was fractured.

Parental concerns that buddy taping might be insufficient because children can remove it by themselves are unfounded. Although patient compliance with activity restriction by splinting in children varies (Cornwall and Ricchetti, 2006), we found that buddy taping was well tolerated, indicating higher comfort levels than did splinting. However, differences of 1.6 at 5 days and 1.3 at 21 days may not be clinically relevant. By contrast to other studies (Rashid et al, 2005; Won et al., 2014), we found no skin-related complications such as skin necrosis, probably because interdigital padding was used and parents received instruction on how to change a taping. The dynamic immobilization provided by taping might be insufficient for pain relief in some oblique fractures (Nellans and Chung, 2013; Weber et al., 2019). One patient with an oblique fracture required a change from taping due to persistent pain after 5 days. However, no statistical group differences were found in post-interventional pain scores and duration of analgesic medication.

This study has limitations. To ensure a sample of sufficient size to provide adequate statistical power, we included all displaced finger fractures from our initial study, which used the same study protocol. The partial re-use of data from our previous study, collected from 31 displaced fractures requiring reduction, does not pose a multiple testing issue because a predefined strict fixed sequence procedure was followed (Huque and Alosh, 2008). Additionally, phalangeal neck fractures were considered inherently unstable and therefore excluded from the study. Due to the small number of oblique fractures included in our sample, we cannot ascertain that taping always provides sufficient analgesia for this fracture type.

Our previous study demonstrated that taping can be recommended for all undisplaced proximal and middle phalangeal finger fractures except intra-articular and phalangeal neck fractures in children. With the current data, we can conclude that taping these finger fractures can be recommended irrespective of the degree of displacement or the need for reduction. The higher patient comfort, lower cost and shorter application time encourage us to propose taping as an alternative to splinting.

## Supplemental Material

sj-pdf-1-jhs-10.1177_17531934241293338 - Supplemental material for Buddy taping after reduction of displaced extra-articular phalangeal finger fractures in children: a randomized controlled trial Supplemental material, sj-pdf-1-jhs-10.1177_17531934241293338 for Buddy taping after reduction of displaced extra-articular phalangeal finger fractures in children: a randomized controlled trial by Daniel M. Weber, Christian Luckert, Markus Kalisch, Ulrike Subotic, Robert Weil and Michelle Seiler in Journal of Hand Surgery (European Volume)

sj-pdf-2-jhs-10.1177_17531934241293338 - Supplemental material for Buddy taping after reduction of displaced extra-articular phalangeal finger fractures in children: a randomized controlled trial Supplemental material, sj-pdf-2-jhs-10.1177_17531934241293338 for Buddy taping after reduction of displaced extra-articular phalangeal finger fractures in children: a randomized controlled trial by Daniel M. Weber, Christian Luckert, Markus Kalisch, Ulrike Subotic, Robert Weil and Michelle Seiler in Journal of Hand Surgery (European Volume)

sj-pdf-3-jhs-10.1177_17531934241293338 - Supplemental material for Buddy taping after reduction of displaced extra-articular phalangeal finger fractures in children: a randomized controlled trial Supplemental material, sj-pdf-3-jhs-10.1177_17531934241293338 for Buddy taping after reduction of displaced extra-articular phalangeal finger fractures in children: a randomized controlled trial by Daniel M. Weber, Christian Luckert, Markus Kalisch, Ulrike Subotic, Robert Weil and Michelle Seiler in Journal of Hand Surgery (European Volume)

## Trial registration

ClinicalTrials.gov NCT04068935
