# Supplementary material for: Buddy taping after reduction of displaced extra-articular phalangeal finger fractures in children: a randomized controlled trial
Source: J Hand Surg Eur Vol. 2024 Nov 2;50(5):622–7. doi: 10.1177/17531934241293338 (PMC12012284; doi:10.1177/17531934241293338)
Supplement: sj-pdf-1-jhs-10.1177_17531934241293338 - Supplemental material for Buddy taping after reduction of displaced extra-articular phalangeal finger fractures in children: a randomized controlled trial [file sj-pdf-1-jhs-10.1177_17531934241293338.pdf]

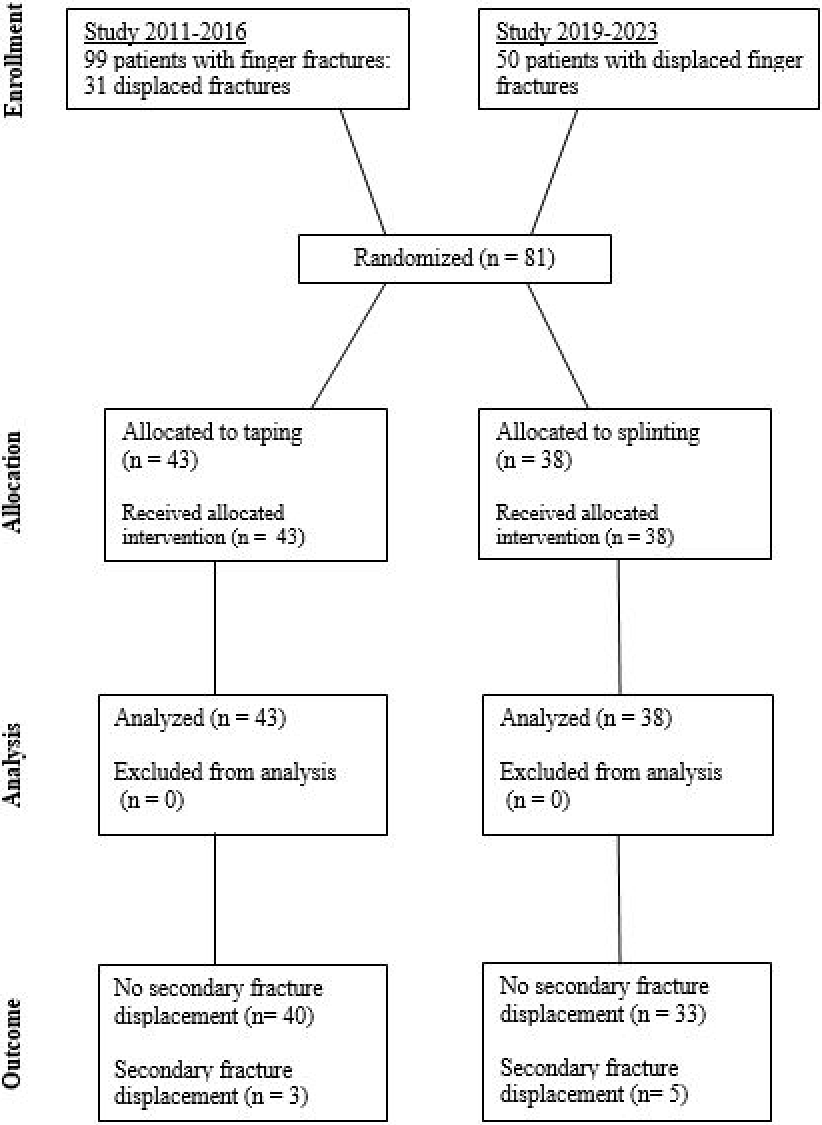
Enrollment
Study 2011-2016
99 patients with finger fractures:
31 displaced fractures
Study 2019-2023
50 patients with displaced finger fractures
Randomized (n = 81)
Allocation
Allocated to taping (n = 43)
Received allocated intervention (n = 43)
Allocated to splinting (n = 38)
Received allocated intervention (n = 38)
Analysis
Analyzed (n = 43)
Excluded from analysis (n = 0)
Analyzed (n = 38)
Excluded from analysis (n = 0)
Outcome
No secondary fracture displacement (n= 40)
Secondary fracture displacement (n = 3)
No secondary fracture displacement (n = 33)
Secondary fracture displacement (n= 5)
