# Supplementary figures and images for: Buddy taping after reduction of displaced extra-articular phalangeal finger fractures in children: a randomized controlled trial
Source: J Hand Surg Eur Vol. 2024 Nov 2;50(5):622–7. doi: 10.1177/17531934241293338 (PMC12012284; doi:10.1177/17531934241293338)

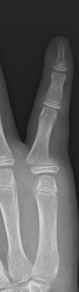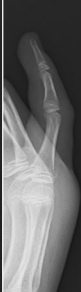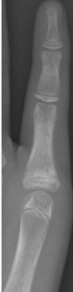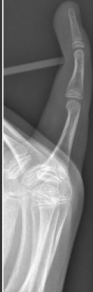

Supplement: sj-pdf-2-jhs-10.1177_17531934241293338 - Supplemental material for Buddy taping after reduction of displaced extra-articular phalangeal finger fractures in children: a randomized controlled trial [file sj-pdf-2-jhs-10.1177_17531934241293338.pdf]

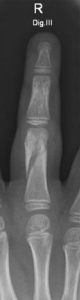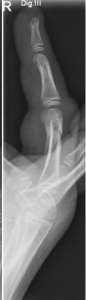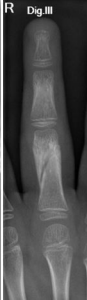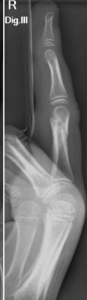

Supplement: sj-pdf-3-jhs-10.1177_17531934241293338 - Supplemental material for Buddy taping after reduction of displaced extra-articular phalangeal finger fractures in children: a randomized controlled trial [file sj-pdf-3-jhs-10.1177_17531934241293338.pdf]
